# Sustainable continuous renal replacement therapy: The influence of blood flow rates, effluent dose, autoeffluent, and citrate anticoagulation on carbon dioxide emissions

**DOI:** 10.1016/j.ccrj.2026.100176

**Published:** 2026-04-19

**Authors:** Cyveen Weeraratna, Forbes McGain, Andrew A. Udy, Emily J. See, Christian Karcher, Patrick R. Joyce, Scott McAlister, Catherine J. O'Shea, Mayuri G.W. Wijayasundara, Natalie Adams, Susan R. James, Benjamin W. Sansom

**Affiliations:** a Intensive Care Unit, Royal Melbourne Hospital, Victoria, Australia; b Intensive Care Unit, Monash Children's Hospital, Victoria, Australia; c Department of Critical Care, Faculty of Medicine, Dentistry and Health Sciences, University of Melbourne, Australia; d Intensive Care Medicine, Western Health, Melbourne, Victoria, Australia; e Intensive Care Medicine, Alfred Hospital, Melbourne, Victoria, Australia; f Alfred Intensive Care, Melbourne, Victoria, Australia; g Monash University, Melbourne, Victoria, Australia; h Intensive Care Medicine - Royal Melbourne Hospital, Victoria, Australia; i Royal Melbourne Hospital, Victoria, Australia; j University of Melbourne, Australia; k Monash University, Australia; l University of Melbourne, Victoria, Australia; m Donate Life, Victoria, Australia; n Intensive Medicine, Alfred Hospital, Melbourne, Victoria, Australia; o Alfred Hospital, Melbourne, Victoria, Australia; p Adjunct Senior Research Fellow, School of Public Health and Preventative Medicine, Monash University, Australia; q Healthcare Sustainability, University of Melbourne, Victoria, Australia; r Deakin University, Sustainability and Circular Economy, Australia; s Anvarta Pty Ltd, Australia; t Intensive Care Unit, Alfred Hospital, Melbourne, Victoria, Australia; u Intensive Care, Alfred Hospital, Melbourne, Victoria, Australia; v Royal Melbourne Hospital, Australia

**Keywords:** Continuous renal replacement therapy (CRRT), Carbon dioxide equivalent (CO_2_-e), Life cycle analysis (LCA), Sustainability in intensive care, Consumables

## Abstract

**Objectives:**

The aim of this study was to quantify the consumables waste for continuous renal replacement therapy (CRRT) in intensive care, determine the carbon dioxide equivalent (CO_2_e) per patient per day, and identify the impact of blood (Q_b_) and effluent flow rates, citrate anticoagulation, autoeffluent, and circuit lifespan on emissions and cost.

**Design:**

A retrospective observational study was conducted.

**Setting:**

The study included two major tertiary intensive care units in Melbourne from January 2017 to December 2024.

**Participants:**

We analysed 70,000 h of treatment data stored on CRRT machines, captured from 2464 patients.

**Interventions:**

Quantities of all major consumables were obtained. A chemical composition analysis was performed, and life cycle analysis was done for each component.

**Main outcome measures:**

Carbon dioxide–equivalent values and financial cost estimates were calculated using local, commercial pricing.

**Results:**

Combined impact of autoeffluent, lowest effluent rate (<30 mL/kg/hr), and doubling of filter life linked to Q_b_ (130–150 mL/h) and citrate anticoagulation was associated with a 56.8% [54.4%–59.1%] decrease in CO_2_e.kg/day and 38.1% [34.8%–40.9%] cost reduction.

Median CO_2_e.kg/day fell from 25.3 [21.5–29.7] in 2017 to 19.3 [16.3 – 22.8] in 2024, p <0.0001. The total cost per day of consumables also fell from $995.89 ($820.40 – $1210.67) in 2017 to $829.07 ($689.64 – $1069.19) in 2024.

**Conclusion:**

Extended circuit lifespan, autoeffluent, and moderated effluent doses translate to the most sustainable delivery of CRRT with associated cost savings.

## 1 Introduction

Health care accounts for 5% of Australia’s greenhouse gas emissions,^1^ and intensive care generates high volumes of consumables waste, contributing to carbon emissions and costs.^2^ Environment, social, and governance reporting requirements of health services demand strong data-led strategies to drive this figure down to reduce health care’s impact on climate change. As such, comprehensive analyses of consumables waste are important to inform decision-making at manufacturing, procurement, and clinical levels.

Renal replacement therapy is recognised as a significant contributor to consumables waste in the healthcare setting. In patients with chronic kidney disease, intermittent haemodialysis alone is estimated to produce 1.5 billion tonnes of waste annually, more than any other healthcare procedure.^3^ Nephrologists have reported the substantial environmental effects of long-term renal support for the last two decades.^4^ In response, programs such as the National Health Service’s Sustainable Healthcare Programme have achieved annual savings of 10 million euros through improvements in water sterilisation efficiency and reduced electricity usage.

Unlike intermittent haemodialysis, the environmental burden of continuous renal replacement therapy (CRRT) is not well understood, despite being delivered to 8000 intensive care unit (ICU) patients in Australia and New Zealand per year (Appendix 1, Table A1 – ANZICS CRRT and total admissions data). Accordingly, we aimed to quantify major consumables during CRRT, estimate the associated carbon emissions and costs, and measure the impact of (i) filter life; (ii) blood flow and effluent flow rates; (iii) citrate anticoagulation; and (iv) use of an automatic effluent drainage system (auto-effluent). We used benchmarks derived from current clinical evidence around timing of initiation, dialysis dose, citrate anticoagulation, and blood flow rates, with cost and carbon dioxide emissions presented alongside the evolution of practice over time. We hypothesised that lower effluent rates, use of citrate anticoagulation, longer filter life, and use of auto-effluent were likely to reduce financial costs and carbon emissions.

## 2 Methods

### 2.1 Design

We undertook a retrospective observational study evaluating the major consumables waste generated during CRRT for intensive care patients at two large tertiary ICUs in Melbourne from January 2017 to December 2024 and estimated the carbon impact and cost.

### 2.2 Participants, devices, and CRRT protocol

**Fig. A1:**
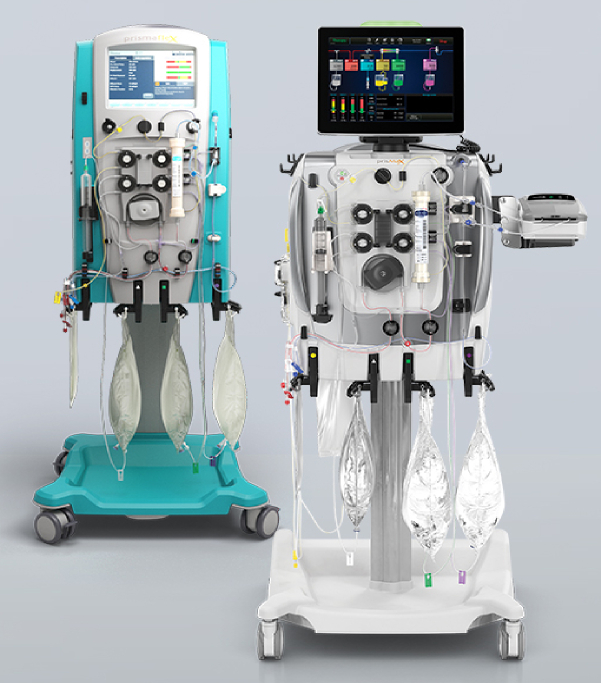
Prismaflex® or PrisMax® device (Vantive, IL, USA) Image reproduced from www.baxter.com Left hand side - Prismaflex®, Right hand side - PrisMax® Each machine connected to circuit with 3 solution bags and 1 effluent collection bag

**Fig. A2:**
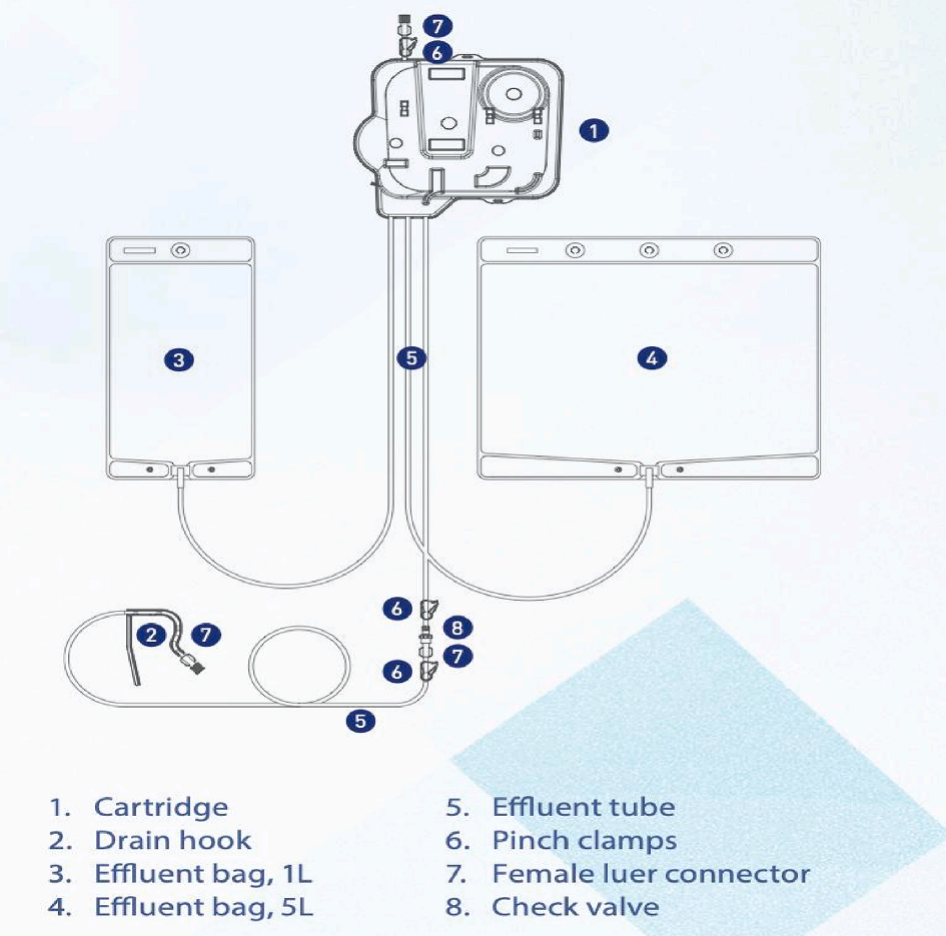
Autoeffluent circuit diagram (courtesy Baxter/Vantive) Autoeffluent circuit as available for *PrisMax®* machine

Between January 2017 and December 2024, we included adult patients undergoing CRRT if they had a recorded treatment on the Prismaflex® or PrisMax® device (Vantive, IL, USA) (Appendix 1, Figure A1 – Images of machines with circuits connected). CRRT was prescribed as continuous venovenous haemodiafiltration (generally a 1:1 dialysis-to-filtration ratio, predominantly predilution) or continuous venovenous haemodialysis, with citrate, heparin, low-molecular-weight heparin, heparin-protamine, or no circuit anticoagulation. Fluid removal (or net ultrafiltration) was at the discretion of the treating clinician, and effluent rate was 25–50 mL/kg/hr. Blood flow (Q_b_) was 150–250 mL/min at hospital A and 130 mL/min at hospital B, the former changing practice to reduced dialysate and blood flow rates in 2022. For citrated circuits, an 18-mmol/L trisodium citrate solution was used as predilution (pre–blood pump) to deliver either 2.7 mmol/L citrate predilution (hospital A) or 3 mmol/L (initially titrated up or down to a postfilter ionised calcium of 0.25–0.4, hospital B). Predilution volume was calculated by the machine whereby it was fixed by Q_b_ and citrate dose. Dialysate utilised a calcium-free bicarbonate-buffered solution. Postdilution was done either with a bicarbonate-buffered solution or with a calcium-free lower bicarbonate solution, generally set to 200 mL/h, and dialysate flow was either the balance to achieve total effluent rate of 30 mL/kg/hr or set to match the total convective volume (i.e., predilution + postdilution). Noncitrate circuits used bicarbonate-buffered solutions as predilution, postdilution, and dialysate. Membranes used were AN69ST100 or AN69ST150 (Vantive, IL, USA). All pre-prepared dialysis solutions were presented in 5-L bags and connected inline to membrane sets. Effluent was discharged either into single-use 5-L collection bags or directly into the hospital sewerage drainage system via an autoeffluent circuit, the latter option only being available with PrisMax® devices (Vantive, IL, USA, Appendix 1, Figure A2 – Autoeffluent circuit diagram).

### 2.3 Variables and data sources

#### 2.3.1 Primary data source and patient data

Prismaflex® or PrisMax® devices (Vantive, IL, USA) record and store comprehensive data for every circuit run, which can be used for quality and training purposes. Parameters for Q_b_, net ultrafiltration, effluent rate, and filter life were transferred from machine data storage and imported into “R” and “RStudio” software (Appendix 1, Table A2 – R packages used), for this study. Each unit record number per patient, entered by nursing staff into machines when commencing CRRT, is encrypted to generate a unique identifier. All CRRT circuit runs per patient are linked through this identifier. Aggregate patient characteristics during the period studied were extracted from the Australian and New Zealand Intensive Care Society (ANZICS) Adult Patient Database (APD) (Table 1 - Patient Characteristics during the period of study from ANZICS Adult Patient Database) and not linked to machine data or unique identifiers.

**Table 1:** Patient characteristics in the study period from ANZICS Adult Patient Database.

Variable	2017	2018	2019	2020	2021	2022	2023	2024
N	343	451	492	465	488	480	497	528
Age, years [95% CI]^a^	60.4 [47.5 to 69.8]	60.2 [47.3 to 70.0]	59.9 [48.3 to 69.6]	61.0 [48.8 to 70.6]	61.8 [49.0 to 71.7]	59.5 [47.3 to 70.2]	61.1 [48.0 to 71.1]	61.0 [48.5 to 69.2]
Males, n (%)^b^	225 (65.6)	304 (67.4)	337 (68.5)	305 (65.6)	310 (63.5)	318 (66.2)	349 (70.2)	361 (68.4)
Hospital A, n (%)^b^	220 (64.1)	318 (70.5)	321 (65.2)	271 (58.3)	302 (61.9)	281 (58.5)	295 (59.4)	315 (59.7)
Length of stay, days [IQR]^c^	7.7 [3.2 to 15.6]	8.3 [3.8 to 15.9]	7.0 [2.9 to 14.8]	7.2 [3.0 to 14.9]	8.0 [3.2 to 17.1]	7.6 [3.1 to 15.0]	7.4 [2.8 to 16.7]	7.8 [3.2 to 16.0]
AP3, ROD [IQR]^c^	41.0 [17.8 to 75.6]	34.6 [15.8 to 66.8]	36.3 [15.8 to 72.6]	35.3 [17.0 to 68.5]	36.5 [16.0 to 71.1]	37.1 [16.7 to 74.2]	32.1 [13.3 to 68.7]	34.5 [13.1 to 66.0]
Mechanical ventilation, n (%)^b^	287 (83.7)	348 (77.2)	352 (71.5)	309 (66.5)	341 (69.9)	315 (65.6)	339 (68.2)	353 (66.9)
Noninvasive ventilation, n (%)^b^	216 (63)	272 (60.3)	274 (55.7)	229 (49.2)	256 (52.5)	258 (53.8)	276 (55.5)	278 (52.7)
Mortality, n (%)^b^	120 (35)	135 (29.9)	156 (31.7)	110 (23.7)	138 (28.3)	134 (27.9)	130 (26.2)	138 (26.1)
Admission diagnosis—surgical^b^								
Cardiothoracic surgery, n (%)	36 (10.5%)	43 (9.5%)	30 (6.1%)	39 (8.4%)	43 (8.8%)	32 (6.7%)	48 (9.7%)	44 (8.3%)
Other surgical, n (%)	17 (5%)	21 (4.7%)	19 (3.9%)	26 (5.6%)	25 (5.1%)	24 (5%)	30 (6%)	20 (3.8%)
Admission diagnosis—medical^b^								
Cardiovascular, n (%)	80 (23.3%)	137 (30.4%)	150 (30.5%)	131 (28.2%)	126 (25.8%)	143 (29.8%)	130 (26.2%)	164 (31.1%)
Haematological, n (%)	10 (2.9%)	10 (2.2%)	4 (0.8%)	8 (1.7%)	11 (2.3%)	7 (1.5%)	8 (1.6%)	6 (1.1%)
Liver failure or transplant, n (%)	4 (1.2%)	5 (1.1%)	3 (0.6%)	6 (1.3%)	6 (1.2%)	6 (1.2%)	6 (1.2%)	7 (1.3%)
Other medical, n (%)	22 (6.4%)	28 (6.2%)	38 (7.7%)	28 (6%)	32 (6.6%)	27 (5.6%)	41 (8.2%)	42 (8%)
Pneumonia, n (%)	20 (5.8%)	10 (2.2%)	7 (1.4%)	13 (2.8%)	41 (8.4%)	35 (7.3%)	15 (3%)	20 (3.8%)
Renal, n (%)	16 (4.7%)	34 (7.5%)	37 (7.5%)	26 (5.6%)	30 (6.1%)	29 (6%)	38 (7.6%)	45 (8.5%)
Respiratory, n (%)	20 (5.8%)	24 (5.3%)	36 (7.3%)	17 (3.7%)	30 (6.1%)	20 (4.2%)	20 (4%)	26 (4.9%)
Sepsis—shock, n (%)	62 (18.1%)	55 (12.2%)	74 (15%)	71 (15.3%)	47 (9.6%)	48 (10%)	51 (10.3%)	49 (9.3%)
Sepsis, n (%)	32 (9.3%)	51 (11.3%)	58 (11.8%)	75 (16.1%)	61 (12.5%)	67 (14%)	72 (14.5%)	76 (14.4%)
Toxicology, n (%)	5 (1.5%)	6 (1.3%)	7 (1.4%)	4 (0.9%)	5 (1%)	11 (2.3%)	8 (1.6%)	5 (0.9%)

ANZICS: Australian and New Zealand Intensive Care Society; CI: confidence interval; IQR: interquartile range.

a Values are presented as mean [95% confidence interval].

b Values are presented as number (percentage of total for that year).

c Values are presented as median [interquartile range].

Test runs and training/demonstration circuits were identified through knowledge of the encrypted unique identifiers for “demo” and “test” entries and removed. Any “patient” where their total therapy time was less than 5 h was also excluded as these were likely also test/training runs.

#### 2.3.2 Materials usage

Total consumables usage was calculated on a per-patient basis and presented per day (24 h) of CRRT delivered. Volumes per CRRT circuit run were divided by 5000 mL (i.e., one bag of dialysate/filtrate) to obtain the number of bags used (and rounded up to the nearest integer) for effluent (where auto-effluent not used), predilution, postdilution, and dialysate. The number of membrane and circuit tubing sets per circuit run was “1”, with set type extracted from machine data. Use of auto-effluent was established from machine events records, and for every 7 days of therapy, it was assumed a new auto-effluent set would be used, as per manufacturer recommendations. This was established as a total number of auto-effluent sets used for a patient. All materials’ usage was then divided by the number of days of treatment.

#### 2.3.3 Compound analysis, life cycle analysis, and carbon emission estimation

**Fig. A3:**
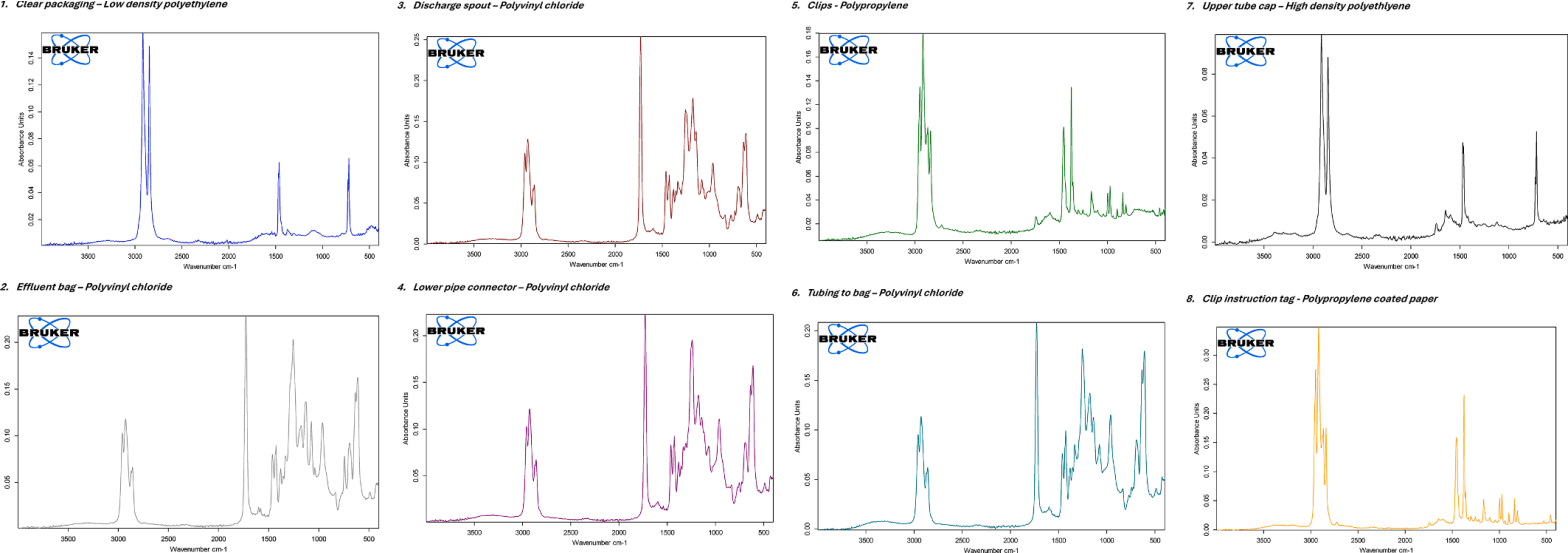
Spectrogram for Prismaflex 5-Litre effluent bag Y axes – Absorbance units; relative peak intensities Individual composition of components detailed in Table A6 presented later, determined from reads-outs mentioned earlier

**Fig. A4:**
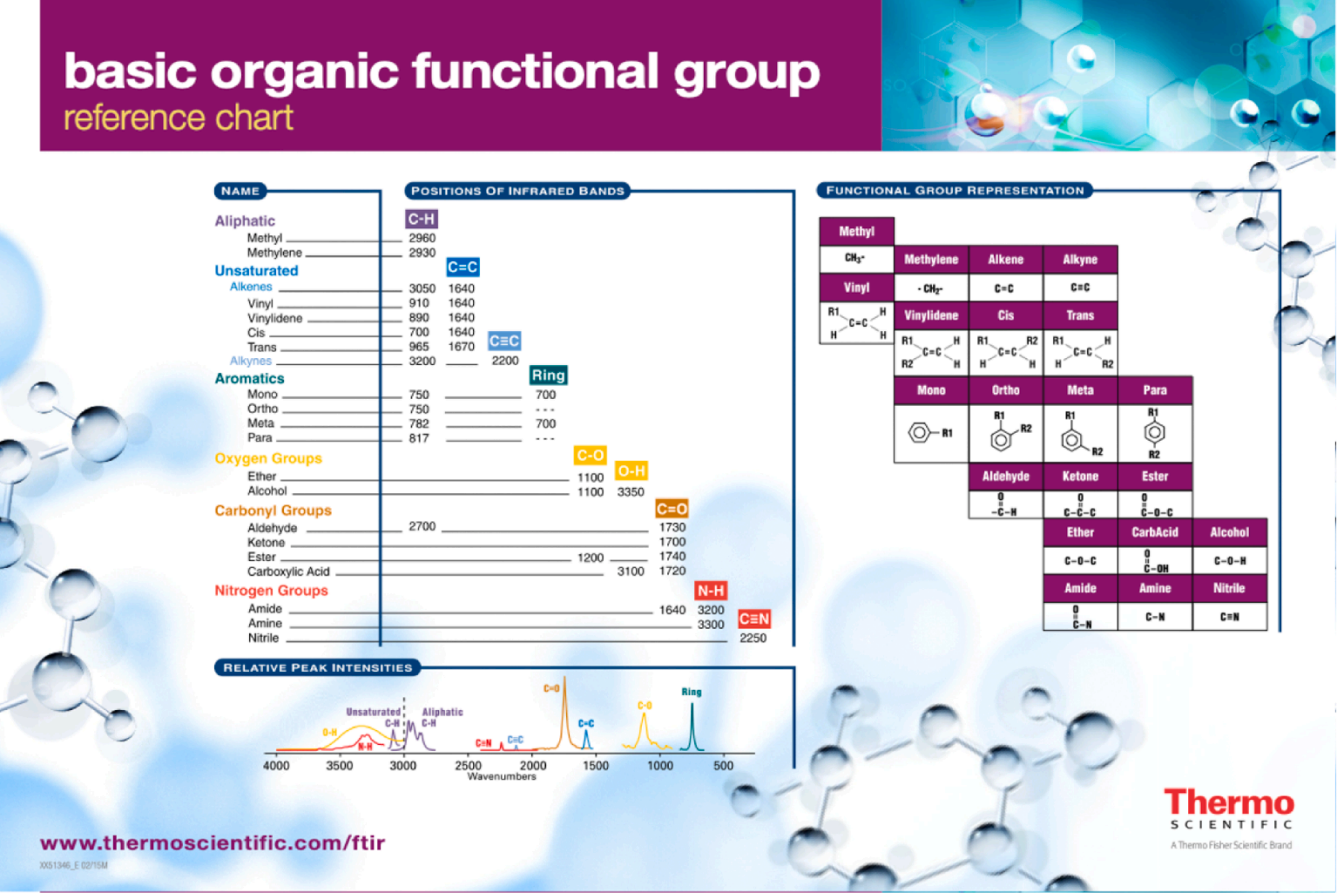
FTIR basic organic functional group reference chart (reproduced from www.thermofisher.com) Industry reference chart for peak absorbances and related functional groups, revealing composite materials. FTIR: Fourier transform infrared

Consumable components were analysed by Fourier transform infrared (FTIR) spectroscopy with attenuated total reflectance at the University of Melbourne to determine absorbance peaks and identify composite compounds, using the Bruker® FTIR attenuated total reflectance device, Germany (Appendix 1, Figure A3/1–8—Spectrogram for Prismaflex 5-Litre effluent bag; Appendix 1, Figure A4 - FTIR Basic Organic Functional Group Reference Chart).

Process-based (PB) life cycle assessment (LCA) quantifies the environmental impact of a product or service throughout its entire life cycle, from raw material extraction to end-of-life disposal. It is a standardised process divided into four key stages: goal and scope definition, life cycle inventory analysis, life cycle impact assessment, and interpretation.[5], [6], [7] Background data on the environmental impacts of materials (e.g., plastics) and manufacture were obtained from the ecoinvent 3.11 APOS database (Appendix 1, Table A3 – Life Cycle Assessment Values).

The functional unit is “Patient dialysis day carbon dioxide equivalent”. Results are in kg carbon dioxide equivalents, using IPCC 2021GWP100 characterisation factors. CO_2_e derives from the emissions related to materials within the bags, extraction, production, transportation, and distribution (wholesale, storage, and delivery to site).^5^

#### 2.3.4 Cost estimation

Local retail purchase pricing in Australian Dollar (AUD) was used as a cost estimate, with each material usage calculated on a per-circuit basis and then per-patient basis. Waste costs were estimated based on state clinical and nonclinical waste pricing, $3.50 (AUD) per kilogram of clinical waste and $0.30 (AUD) per kilogram for nonclinical waste. Hospital waste in the state of Victoria is disposed of via landfill (clinical waste being sterilised prior to disposal) (Appendix 1 – Table A4 - Cost and CO_2_ emissions, unit calculations).

### 2.4 Statistics

Baseline and process observations were summarised as proportions, means (95% confidence interval [CI]), or medians (interquartile range), as appropriate.

Multivariate analyses were performed on a per-patient basis utilising mixed linear modelling with hospital as a random effect. Outcomes of interest were dialysis-day carbon dioxide equivalent (CO_2_e.kg/day) and total cost per day (AUD/day). Variables of interest were use of citrate, use of auto-effluent, approximate effluent rate tertile (<30 mL/kg/hr, 30–40 mL/kg/hr and >40 mL/kg/hr), and log-transformed filter life. Effects were considered significant at a p value <0.005. The sensitivity analysis was performed with a subset of patients who were treated with an effluent rate of 20–25 mL/kg/hr, as per the Kidney Disease Improving Global Outcomes (KDIGO) Clinical Practice Guideline for Acute Kidney Injury. Furthermore, we performed a dose-intensity modelling analysis using mixed-effectlinear regression. Model predictions were generated to obtain expected daily values at effluent doses ranging from 10 to 50 mL/kg/hr.

To estimate the combined effect of multiple predictors, we computed linear contrasts from the fixed effects of the mixed-effect model. Predicted values for the combinations of interest were calculated on the log-transformed scale, and differences were used to derive contrast estimates with corresponding standard errors and 95% CIs based on the model’s variance–covariance structure. We evaluated effect modification using interaction terms between citrate and filter life in linear mixed-effect models. The mediation analysis was performed to decompose total effects of citrate on cost and CO_2_ emissions into direct and indirect (via filter life) pathways, using quasi-Bayesian estimation with 1000 simulations.

### 2.5 Ethical approval

The study was approved as a multisite low-/negligible-risk project (QA2024156) by the local Human Research and Ethics Committee (HREC). Patient consent was not required for this noninterventional retrospective observational study.

## 3 Results

### 3.1 Patient characteristics

A total of 3744 patients underwent CRRT across both sites in the study period. We observed reasonably consistent patient characteristics across the 8-year period (Table 1 – ANZICS Adult Patient Database) with a male predominance and median age of 60 years. This was an expectedly sick cohort of mixed medical/surgical patients with mortality rates between 25 and 35%, often receiving more-than-one organ supports with a prolonged length of stay. Hospital A managed 10–20% more patients requiring CRRT.

### 3.2 Data loss

Of the 3744 patients recorded to have received CRRT, machine-recorded data were available for 2464 patients. Complete information was available for 2017, 2023, and 2024, approximately 80% for 2018 and 2019, and only 25–40% of episodes during 2020–2022. Restricted access to machines by industry during the pandemic, decommissioning of older Prismaflex® devices prior to data access, and software upgrades with failure to save pre-existing data accounted for some data loss. In total, 2464 patients with median circuit lifespans of 25–35 h provided approximately 70,000 h of therapy for analysis and calculations.

### 3.3 Consumable weights, carbon emissions, and cost calculations

The heaviest consumables were the sets themselves (approximately 1 kg) followed by the auto-effluent sets (546 g); while effluent and individual fluid bags were all relatively light (88–139 g), their contributing total weight was due to numbers used (Appendix 1, Table A4). CO_2_ emission estimates/life cycle analyses were derived using the spectrographic analysis shown in Appendix 1, Figures A3.1–A3.8, with Fourier Transform Infrared (FTIR) reference chart Appendix 1, Figure A4, and life cycle analysis values Appendix 1, Table A3. Carbon emissions were the highest per unit for the membrane/tubing sets (6.22–6.95 CO_2_e.kg/day), followed by the auto-effluent devices (2.98 CO_2_e.kg/day), with that for fluid bags being similar around 0.60 CO_2_e.kg/day. Purchase cost contributed most costs, and unit costs were the highest for the membrane/tubing sets followed by the auto-effluent apparatus. Waste cost was the highest for clinical waste elements of consumables (Appendix 1, Table A4).

### 3.4 Consumable usage, carbon emissions, and cost over time and associated circuit variables

**Fig. 1:**
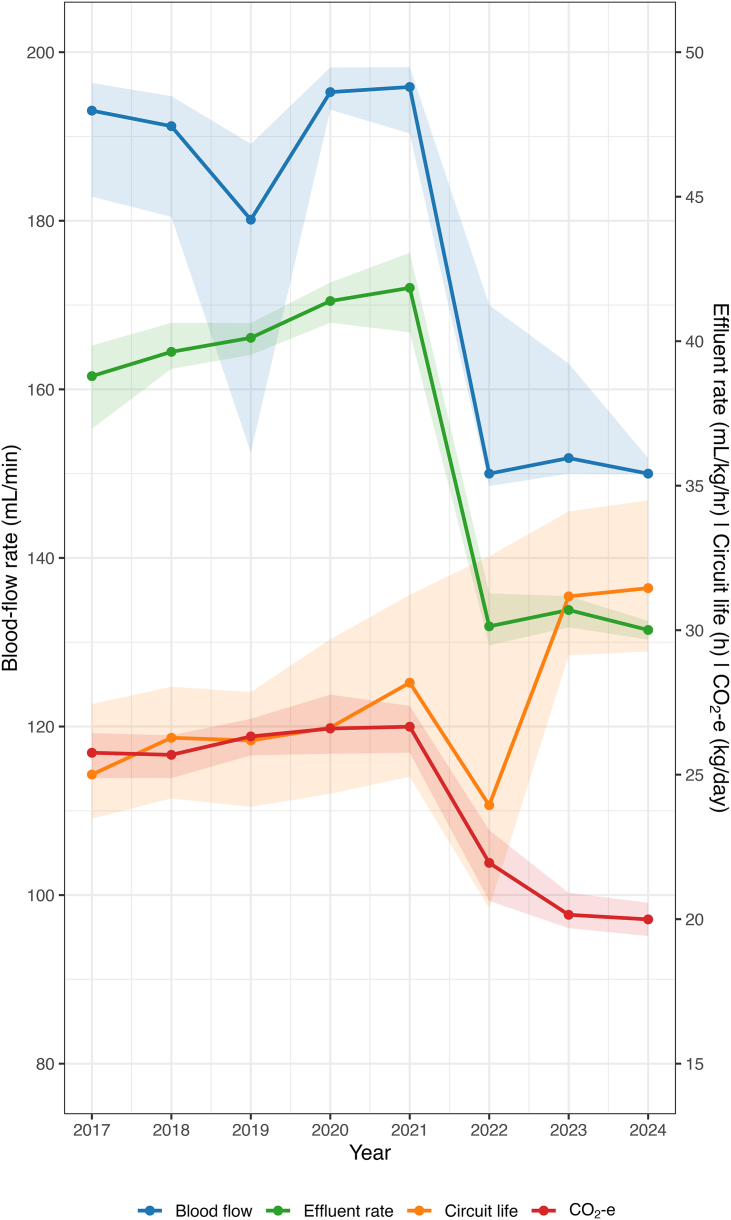
Changes by year, blood flow, effluent rate, circuit life, and CO_2_ emissions Note different Y-axis for blood flow (left) versus effluent rate, circuit life, and CO_2_-e (right). A trend in improved filter life and carbon emissions was seen with reductions in effluent rate and blood pump speed.

Over time, there was a downwards trend of CO_2_ emissions and cost (Fig. 1 and Appendix 1, Table A5). This was associated with use of autoeffluent, reduced effluent rates (predilution and dialysis volumes), and increasing filter life, the latter in turn correlated with lower Q_b_ and increased use of citrate. Median CO_2_e.kg/day fell from 25.3 [21.5–29.7] in 2017 to 19.3 [16.4–22.8] in 2024. The total cost per day of consumables fell in parallel from $995.89 ($820.40–$1210.67) in 2017 to $829.07 ($689.64–$1069.19) in 2024.

### 3.5 Consumable usage, carbon emission, cost, and associated circuit variables by anticoagulation and autoeffluent use

Circuit life values for citrate versus noncitrate circuits without auto-effluent were 32.0 [20.9–44.9] versus 23.7 [14.5–36.6] hours and that with auto-effluent were 34.4 [20.4–50.3] versus 30.7 [18.6–43.5], respectively. Citrate circuits had higher effluent rates, from predilution and dialysate volumes, leading to higher cost and CO_2_ emissions (Table 2). Hospital B used auto-effluent and lower blood flow rates; the former negated the need for effluent bags, reducing waste cost from $10 per day to $5 per day and CO_2_ emissions from approximately 24 CO_2_e.kg/day to 16 CO_2_e.kg/day. Auto-effluent purchase costs outweighed effluent bag costs for short-duration treatments (Table 2).

**Table 2:** Circuit variables, CO_2_ emissions, and cost by citrate and use of autoeffluent.

Variable	Citrate + No autoeffluent	Citrate + Autoeffluent	Noncitrate + No autoeffluent	Noncitrate + Autoeffluent
Total Patients, n	1471	197	699	98
Proportion hospital A, %	90.1	0	63.1	0
Proportion PrisMax, %	36.8	100	29.3	100
Blood flow rate, mL/min	191.0 [150.0–199.5]	129.8 [128.6–130.0]	172.9 [130.0–198.9]	129.6 [126.0–130.0]
Effluent flow rate, mL/kg/hr	38.1 [31.1–43.4]	33.7 [27.9–39.6]	32.4 [27.9–40.9]	30.9 [29.1–35.6]
Net ultrafiltration rate, mL/kg/hr	1.6 [1.1–2.1]	1.1 [0.61–1.6]	1.5 [0.8–2.1]	1.1 [0.3–1.6]
Predilution rate, mL/kg/hr	17.6 [15.7–19.9]	14.1 [11.5–16.4]	13.8 [8.9–16.0]	12.6 [11.5–14.6]
Postdilution rate, mL/kg/hr	2.3 [2.0–2.7]	2.2 [1.8–2.5]	2.4 [2.1–2.8]	2.4 [1.8–2.8]
Dialysis rate, mL/kg/hr	17.6 [9.7–19.3]	16.3 [13.5–18.8]	19.0 [13.2–23.5]	14.8 [14.5–17.4]
Circuit life, hours	32.0 [20.9–44.9]	34.4 [20.4–50.3]	23.7 [14.5–36.6]	30.7 [18.6–43.5]
Predilution bags per day, n	7.6 [6.5–8.0]	6.4 [6.2–6.7]	6.0 [4.0–6.7]	6.2 [4.9–7.5]
Postdilution bags per day, n	1.3 [1.1–1.5]	1.3 [1.1–1.5]	1.4 [1.2–1.9]	1.4 [1.2–1.7]
Dialysis bags per day, n	6.9 [4.6–8.4]	7.4 [7.2–7.8]	7.8 [5.6–9.8]	7.1 [5.9–8.2]
Effluent bags per day, n	15.3 [13.1–17.4]	0.00 [0.00–0.00]	13.7 [11.5–16.1]	0.00 [0.00–0.00]
Sets per day, n	0.7 [0.5–1.1]	0.7 [0.5–1.2]	1.0 [0.7–1.6]	0.8 [0.5–1.3]
Autoeffluent per day, n	0.00 [0.00–0.00]	0.8 [0.5–1.3]	0.00 [0.00–0.00]	0.9 [0.6–1.4]
Predilution cost, $AUD	258.4 [218.3–272.9]	217.0 [211.4–228.2]	158.4 [104.8–180.0]	166.7 [128.3–199.9]
Postdilution cost, $AUD	41.9 [37.0–50.0]	33.2 [28.9–40.0]	38.7 [31.4–50.5]	35.9 [30.5–43.8]
Dialysis cost, $AUD	233.6 [155.5–283.9]	251.3 [243.7–265.3]	209.3 [150.6–260.3]	189.7 [154.5–216.3]
Effluent cost, $AUD	120.74 [102.9–136.8]	0.00 [0.00–0.00]	107.73 [90.3–127.1]	0.00 [0.00–0.00]
Sets cost, $AUD	285.9 [203.7–438.8]	267.7 [183.2–452.5]	385.2 [250.4–622.7]	3 00.3 [212.2–494.6]
Autoeffluent cost, $AUD	0 [0-0]	235.1 [145.1–385.8]	0 [0-0]	251.9 [180.4–409.8]
Waste cost, $AUD	9.8 [8.2–11.5]	4.6 [3.2–6.9]	10.2 [8.2–13.1]	5.0 [3.7–7.7]
Total cost, $AUD	956.0 [800.0–1133.8]	997.7 [845.1–1355.5]	901.2 [708.6–1193.9]	964.6 [766.2–1313.8]
Predilution CO_2_ emissions, CO_2_ e.kg/day	4.6 [4.0–4.9]	3.9 [3.8–4.1]	3.6 [2.4–4.0]	3.7 [2.9–4.5]
Postdilution CO_2_ emissions, CO_2_ e.kg/day	0.8 [0.7–0.9]	0.8 [0.7–0.9]	0.9 [0.7–1.1]	0.8 [0.7–1.0]
Dialysis CO_2_ emissions, CO_2_e.kg/day	4.1 [2.8–5.1]	4.4 [4.3–4.7]	4.7 [3.4–5.9]	4.3 [3.5–4.9]
Effluent CO_2_ emissions, CO_2_e.kg/day	9.2 [7.8–10.4]	0.00 [0.00–0.00]	8.2 [6.9–9.7]	0.00 [0.00–0.00]
Sets CO_2_ emissions, CO_2_e.kg/day	5.2 [3.7–7.8]	4.8 [3.3–8.2]	6.8 [4.5–10.9]	5.4 [3.8–9.0]
Autoeffluent CO_2_ emissions, CO_2_e.kg/day	0.00 [0.00–0.00]	1.1 [0.7–2.2]	0.00 [0.00–0.00]	1.2 [0.7–2.3]
Total CO_2_ emissions, CO_2_e.kg/day	24.2 [20.3–28.1]	15.6 [13.2–19.3]	24.1 [19.8–30.4]	15.9 [12.2–20.4]

All values excluding total numbers and proportions are median [interquartile range].

Waste costs are an aggregate of all consumables waste.

Costs of individual consumable items represent commercial purchase costs.

### 3.6 Impact of effluent rate, anticoagulation, auto-effluent, and filter life on CO_2_ emissions and cost

**Fig. 2:**
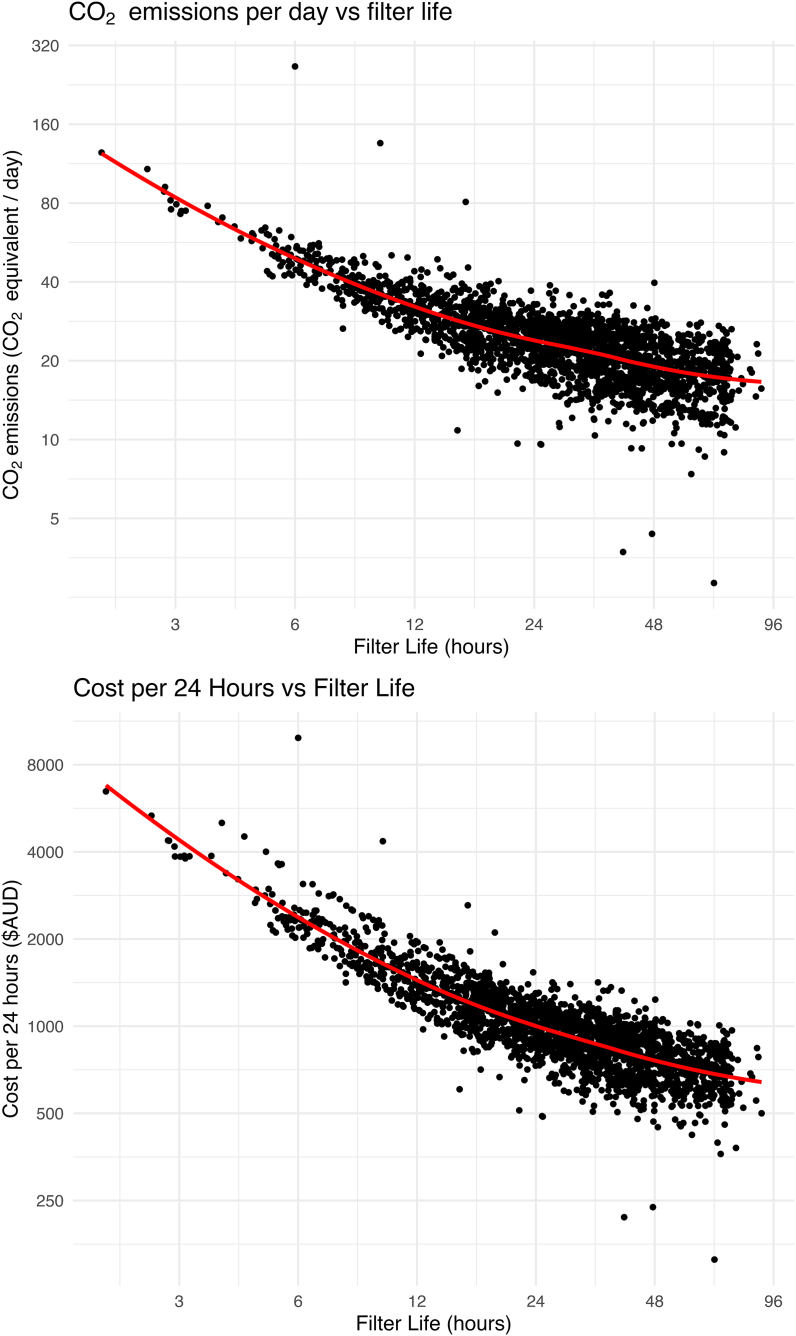
Association of filter life with reduced CO_2_ emissions and cost per day of treatment Cost/day and CO_2_eq.kg/day showed log linear relationship; note both scales are logarithmic for both plots. Each data point represents a patient total cost divided by treatment days and CO_2_eq.kg divided by treatment days. Locally estimated scatterplot smoothing (LOESS) curve has been applied to each.

Associations between anticoagulation, auto-effluent, and effluent rate tertile versus CO_2_ emissions and cost are shown in violin plots (Appendix 1, Fig. 5A–F); additionally, a log linear trend was observed between filter life and both CO_2_ emissions and cost (Fig. 2).

The multivariate analysis revealed auto-effluent and longer filter life correlated with reductions in CO_2_-e of 22.2% [20.2%–24.2%] and 34.3% [33.5%–35.0%], respectively (Table 3), while citrate use and higher effluent rates demonstrated increases in CO_2_e of 5.4% [3.8%–7.0%] and 18.3% [16.2%–20.4%], respectively. Prolonged filter life provided a cost reduction of 42% [40.4%–42.6%], with increased costs linked to citrate (15.8% [12.5%–15.8%]), auto-effluent (23.8% [20.9%–26.7%]), and higher effluent rates (>40 versus <30 mL/kg/hr) 15.6% [13.7%–17.4%]). Combination of auto-effluent, lowest effluent tertile (<30 mL/kg/hr), and doubling of filter life gave a 56.8% [54.4%–59.1%] decrease in CO_2_e.kg/day and a reduced cost of 37.9% [ 34.8%–40.9%]; however, the greatest cost benefit was with no auto-effluent, lowest effluent tertile, and longer filter life, 49.8% [48.5%–51.1%], all p < 0.0001. Citrate anticoagulation was associated with lower CO_2_ emissions and mixed effects on cost, driven by opposing direct and indirect pathways. As anticipated, a strong interaction between citrate and filter life was observed within the CO_2_ and cost models. The mediation analysis confirmed that citrate reduced CO_2_ and cost indirectly via filter life extension, but these gains were partly offset by direct increases in CO_2_ and cost attributable to citrate (Appendix 1, Table A6).

**Table 3:** Mixed linear models: Anticoagulation, autoeffluent, effluent rate tertile, and filter life associations with CO_2_ emissions and cost.

Variable	CO_2_-equivalent effect estimate	Interpretation	p value	Cost effect estimate	Interpretation	p value
Anticoagulation: citrate^a^	1.054 [1.038–1.070]	↑ 5.4% increase in CO_2_e.kg/day	<0.0001	1.158 [1.142–1.175]	↑ 15.8% increase in cost/day	<0.0001
Autoeffluent^b^	0.778 [0.758–0.798]	↓ 22.2% decrease in CO_2_e.kg/day	<0.0001	1.238 [1.209–1.267]	↑ 23.8% increase in cost/day	<0.0001
Effluent rate: 30–40 mL/kg/hr^c^	1.074 [1.056–1.092]	↑ 7.4% increase in CO_2_e.kg/day	<0.0001	1.061 [1.045–1.078]	↑ 6.1% increase in cost/day	<0.0001
Effluent rate: >40 mL/kg/hr^c^	1.183 [1.162–1.204]	↑ 18.3% increase in CO_2_e.kg/day	<0.0001	1.156 [1.137–1.174]	↑ 15.6% increase in cost/day	<0.0001
Filter life^d^	0.657 [0.650–0.665]	↓ 34.3% decrease in CO_2_e.kg/day	<0.0001	0.580 [0.574–0.586]	↓ 42% decrease in cost/day	<0.0001
Combination of autoeffluent^a^, Effluent rate <30 mL/kg/hr^e^, filter life^d^	0.432 (0.418–0.446)	↓ 56.8% decrease in CO_2_e.kg/day	<0.0001	0.621 [0.604 to 0.640]	↓ 37.9% decrease in cost/day	<0.0001

All results were statistically significant (p < 0.0001).

a Versus noncitrate anticoagulation.

b Versus no autoeffluent/effluent bags.

c Versus <30 mL/kg/hr (lowest tertile).

d Estimate is effect for each doubling of filter life.

e Versus >40 mL/kg/hr.

An additional sensitivity analysis of patients receiving 20–25 mL/kg/hr showed a lower overall cost, $923 [769–1292], and CO_2_-e, 22.1 [18.6–28.6] kg/day.

Predicted cost and CO_2_e rose progressively with increasing effluent dose (Appendix A1, Table A8)., An estimated daily consumable cost of approximately $816–$867 per patient-day (95% CI ∼ $410–$1650) and CO_2_ emissions of 22–24 kg CO_2_e per day (95% CI ∼ 13–40 kg) was demonstrated for doses of 20-25mL/kg/hr. At the lowest modelled dose of 10 mL/kg/hr, both cost and CO_2_ were roughly 15–20% lower, whereas at the highest dose of 50 mL/kg/hr, they were 20–25% higher than baseline.

## 4 Discussion

### 4.1 Key findings

This comprehensive analysis of consumable waste with CRRT highlights several key findings. In 2024, CRRT delivered for 24 h translated to a total of 19.3 kg [16.4–22.8] CO_2_e. The membrane and tubing sets accounted for the highest CO_2_e, followed by auto-effluent, and purchase cost dominated total cost. Between 2017 and 2024, there was a downward trend in both CO_2_e and cost. This is most likely explained by focused adjustments to CRRT prescriptions over the same time (Q_b_, lower effluent dose, citrate anticoagulation, and increased auto-effluent), which align with the clinical evidence base and are plausibly associated with reduced waste, cost, and CO_2_e.

### 4.2 Relationship to previous studies

Previous studies have not specifically quantified the burden of consumables waste attributable to CRRT. However, data are available for comparison in related settings. The daily environmental footprint of an ICU patient is estimated at 138 kg CO_2_e in the United States^8^ and 61 kg CO_2_e in France.^9^ This figure is even higher in critically ill patients with sepsis, at 178 kg CO_2_e/day in the US and 88 kg CO_2_e/day in Australia.^4^ When using PB LCA, the highest contributors to carbon emissions are consumables and energy consumption.^10^

From our data, it appears that important modifications in CRRT practice and prescription have translated into related benefits in terms of waste and cost. Studies in support of deferred initiation of CRRT and controlled effluent rates uniformly demonstrate the lack of mortality benefit using more liberal measures.[11], [12], [13], [14], [15], [16], [17], [18] Citrate anticoagulation benefits circuit lifespan,^19^^,^^20^ as does a lower Q_b_,^21^ through reduced access dysfunction, resulting in fewer circuit changes and lower predilution fluids volumes. These collectively translate to reduced waste and cost. Our analysis highlights the parallel impact on reduced carbon emissions through implementing this evidence base. The BEST study found that limiting effluent doses alone to 25 mL/kg/hr has the effect of reducing fluid costs in CRRT by approximately 43.3%,^22^ but in contrast, we found only found a <10% cost reduction.

### 4.3 Implications

Application of sustainability principles of reduce, recycle, reuse, and repurpose assists with improving the overall carbon footprint of CRRT. Dialysis is the highest consumables waste generator in health care.^3^^,^^23^ Baxter Healthcare (now Vantive) identified that 78% of CO_2_ emissions for CRRT was associated with the supply chain of fluid bags, 49% being linked to transport. The products we analysed were all manufactured overseas. Changing the point of manufacture to Australia or Asia (India or China) could be considered. Problematically, their primary fuel source for electricity generation are fossil fuels, compared with Italy and the rest of Europe using predominantly renewables and nuclear.^24^

Recycled polypropylene is more expensive than the virgin product, owing to cost of separation and sterilisation, is less robust and stable with each recycling process, and has higher CO_2_ emissions (Appendix 1, Table A7 - Chemical composition of consumables). Product manufacture using polyvinyl chloride (PVC) facilitates recyclability of all nonhazardous plastic waste. Every tonne of recycled PVC replaces about one tonne of virgin PVC compound in new products, consuming 80% less energy and reducing carbon emissions,^24^ in line with national climate action objectives. In Victoria in 2024, 1818 patients received CRRT, which produced 1.2 kg, per patient dialysis day, of nonhazardous waste, translating to upwards of 2 tonnes of potentially recycled PVC that would avoid landfill and the production of virgin plastic (Appendix 1, Table A1 - ANZICS total admissions and CRRT numbers per year).

While not included in our analysis, cardboard boxes transporting all consumables need to be addressed more broadly. For CRRT alone, between these two hospitals in 2024, 3000–4000 boxes were discarded.

**Fig. 3:**
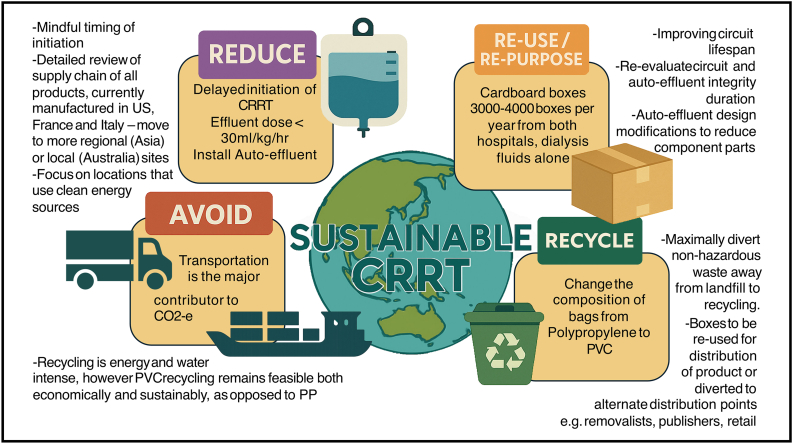
Change management in sustainable CRRT. CRRT: continuous renal replacement therapy.

Refer to Fig. 3 for some focus points of change management in the transition to the sustainable practice of CRRT.

### 4.4 Strengths and limitations

This large data set of 70,000 h over 8 years, enabled identification of associations between different factors surrounding the delivery of CRRT and CO_2_ emissions. Filter life has been previously assumed to benefit resource usage, but this is the first study to demonstrate that prolonged filter life is associated with reduced costs and carbon emissions. The median values of consumables waste will be representative for CRRT in most Australian ICUs, and therefore, CO_2_e and cost data will have local applicability. The patterns will be externally valid, but absolute values will vary per region.

This analysis singularly used Vantive products, which hold a 70% market share of Australia and New Zealand ICUs, creating commercial bias with these results. The alternative companies, B. Braun Avitum AG (OMNI ® and Diapact®, Melsungen, Germany) and Fresenius (multiFiltratePRO® Bad Hornburg, Germany), manufacture fluid bags from almost identical raw materials, and the volumes of waste are likely equivalent to that of Vantive-run therapies as fluid volumes used are similar.

The centre that exclusively uses auto-effluent also uses lower blood flow rates, whereby the impact auto-effluent independently makes on circuit lifespan and waste may be amplified.

Our PB LCA introduces a risk of truncation bias compared with economic input–output LCA; however, the former provides relevant and interpretable information. This retrospective observational study poses the risk of confounders and therefore limits causal interpretation. Significant data loss, with the data obtained over 2020–2022, predominantly from hospital A, may affect overall validity of findings; however, the sheer volume of data likely mitigates this. Some data were rejected for potentially representing test or training runs, deemed advantageous for integrity of the dataset on balance. We also did not look at the comparative CO_2_e of providing intermittent haemodialysis in the ICU, which is a feasible clinical alternative, and potentially both carbon and cost saving.

## 5 Conclusions

CRRT is associated with significant generation of consumable waste, although the overall burden has decreased over time. Cohorted use of auto-effluent, a lower effluent rate (<30 mL/kg/hr), and longer treatment duration (every doubling of filter life) were associated with a 56.8% reduction in CO_2_e per 24 h (estimate: 0.397 [0.365–0.427]) compared to the reference group, a combination that may significantly reduce environmental impact. This is potentially mediated by low blood flow rates and citrate anticoagulation. Sustainably delivered CRRT in the ICU is a realistic vision for the near future.

## Conflict of interest

The authors declare the following financial interests/personal relationships which may be considered as potential competing interests: Co-authors Associate editor—Prof Andrew A. Udy, Alfred Hospital Intensive Care Unit—Consultant and Head of research. Monash University Australia and New Zealand Intensive Care Research Centre, Boonwurrung Country Victoria, Australia Editorial board—Dr Emily See, Royal Melbourne Hospital Intensive Care Unit and Department of Nephrology Parkville, Victoria, Australia—Consultant Adjunct senior research fellow—Monash University Senior Honorary Fellow—University of Melbourne. If there are other authors, they declare that they have no known competing financial interests or personal relationships that could have appeared to influence the work reported in this paper. There were no external sources of funding for this rese

## CRediT authorship contribution statement

1. Dr Benjamin W. Sansom, Senior Intensive Care Registrar, Royal Melbourne Intensive Care Unit – data interrogation, statistical analysis, editing of manuscript, mentorship.

2. Ms Catherine J. O'Shea, Program Manager of Healthcare Carbon Lab, University of Melbourne – composition analysis of each component of consumables.

3. Dr Scott McAlister, University of Melbourne, Senior Research Officer–Life cycle analysis of each component of consumables.

4. Dr Mayuri G.W. Wijayasundara, Honorary Fellow Deakin University, CEO Anvarta Pty Ltd. - Expertise on sustainability, circular economy and change management.

5. A/Professor Forbes McGain, Associate Dean Healthcare Sustainability, University of Melbourne – subject expertise, guidance of carbon emissions calculations.

6. Professor Andrew A. Udy – Consultant Intensivist and Head of Research Alfred hospital – mentorship for initiation of project.

7. Dr Emily J. See – Consultant Intensivist and Nephrologist Royal Melbourne Hospital and Senior Honorary Fellow, University of Melbourne and Adjunct Senior Research Fellow, Monash University – project supervisor.

8. Dr Patrick R. Joyce – Consultant Intensivist and ICU Renal clinical lead Alfred Hospital – supervisor for ethics application at Alfred hospital.

9. Dr Christian Karcher – Consultant Intensivist Royal Melbourne Hospital – Supervisor of training.

10. Ms Natalie Adams – Clinical Nurse Consultant, Renal Support Therapies, Intensive Care Unit, Alfred Hospital – assistance with data retrieval.

11. Ms Susan R. James – Critical Care Registered Nurse, Sustainability lead, Intensive care unit, Alfred Hospital – assistance with communication with commercial recycling companies and environmental services at Alfred Hospital.

12. Mrs Megan Taylor – Vantive Product Specialist – Assistance with data retrieval and provisions of consumables for analysis.

## Figures and Tables

**Table A1:** Total ICU admissions, number of patients receiving CRRT, and percentage receiving CRRT per state per year, between 2017 and 2024.

State	Year	Total	CRRT	Percentage
VIC	2017	41,089	735	0.02
VIC	2018	43,563	1490	0.03
VIC	2019	44,790	1668	0.04
VIC	2020	42,665	1840	0.04
VIC	2021	43,878	1868	0.04
VIC	2022	41,877	1828	0.04
VIC	2023	43,690	1777	0.04
VIC	2024	45,990	1818	0.04
ACT	2017	4495	127	0.03
ACT	2018	4721	175	0.04
ACT	2019	4943	174	0.04
ACT	2020	4927	148	0.03
ACT	2021	5024	185	0.04
ACT	2022	4752	192	0.04
ACT	2023	5040	218	0.04
ACT	2024	5322	221	0.04
NSW	2017	56,498	442	0.01
NSW	2018	59,315	1480	0.02
NSW	2019	64,217	1741	0.03
NSW	2020	64,989	1706	0.03
NSW	2021	65,409	2084	0.03
NSW	2022	62,843	2156	0.03
NSW	2023	65,896	2367	0.04
NSW	2024	61,870	2001	0.03
NT	2017	1858	51	0.03
NT	2018	1742	218	0.13
NT	2019	1771	200	0.11
NT	2020	1721	241	0.14
NT	2021	1676	176	0.11
NT	2022	1706	170	0.10
NT	2023	1679	166	0.10
NT	2024	1744	171	0.10
QLD	2017	29,346	465	0.02
QLD	2018	31,225	1034	0.03
QLD	2019	32,402	1277	0.04
QLD	2020	31,112	1215	0.04
QLD	2021	31,217	1455	0.05
QLD	2022	30,529	1476	0.05
QLD	2023	32,177	1441	0.04
QLD	2024	31,366	1494	0.05
SA	2017	12,513	73	0.01
SA	2018	13,403	302	0.02
SA	2019	14,606	370	0.03
SA	2020	15,475	357	0.02
SA	2021	17,443	381	0.02
SA	2022	17,726	451	0.03
SA	2023	17,764	406	0.02
SA	2024	16,396	421	0.03
TAS	2017	3187	18	0.01
TAS	2018	3584	104	0.03
TAS	2019	3542	99	0.03
TAS	2020	2259	84	0.04
TAS	2021	2746	115	0.04
TAS	2022	3210	129	0.04
TAS	2023	3420	158	0.05
TAS	2024	2183	112	0.05
WA	2017	12,001	53	0.00
WA	2018	12,981	408	0.03
WA	2019	14,115	552	0.04
WA	2020	14,277	551	0.04
WA	2021	14,366	605	0.04
WA	2022	12,979	614	0.05
WA	2023	13,847	686	0.05
WA	2024	14,399	685	0.05
Total		1,411,223	47,491	0.03

Data obtained from ANZICS CORE database, revealing total numbers of patients admitted to the ICU per year and those registered to have received CRRT: 0.03% of ICU patients receive CRRT in Australia.

Transition of databases occurred in 2016/17.

ANZICS: Australian and New Zealand Intensive Care Society; CRRT: continuous renal replacement therapy; ICU: intensive care unit; VIC, Victoria; ACT, Australian Capital Territory; NSW, New South Wales; NT, Northern Territory; QLD, Queensland; SA, South Australia; TAS, Tasmania; WA, Western Australia; ANZICS CORE, Australia and New Zealand Intensive Care Society Centre for Outcome and Resource Evaluation.

**Table A2:** R Packages used

Package	Reference
broom.mixed	Bolker B, Robinson D (2024). _broom.mixed: Tidying Methods for Mixed Models_. R package version 0.2.9.6, <https://CRAN.R-project.org/package=broom.mixed>.
data.table	Dowle M, Srinivasan A (2023). _data.table: Extension of “data.frame”_. R package version 1.14.8, <https://CRAN.R-project.org/package=data.table>.
Dbplyr	Wickham H, Girlich M, Ruiz E (2023). _dbplyr: A “dplyr” Back End for Databases_. R package version 2.3.2, <https://CRAN.R-project.org/package=dbplyr>.
Dplyr	Wickham H, François R, Henry L, Müller K, Vaughan D (2023). _dplyr: A Grammar of Data Manipulation_. R package version 1.1.2, <https://CRAN.R-project.org/package=dplyr>.
Forcats	Wickham H (2023). _forcats: Tools for Working with Categorical Variables (Factors)_. R package version 1.0.0, <https://CRAN.R-project.org/package=forcats>.
ggplot2	H. Wickham. ggplot2: Elegant Graphics for Data Analysis. Springer-Verlag New York, 2016.
Ggpubr	Kassambara A (2023). _ggpubr: ‘ggplot2’ Based Publication Ready Plots_. R package version 0.6.0, <https://CRAN.R-project.org/package=ggpubr>.
gridExtra	Auguie B (2017). _gridExtra: Miscellaneous Functions for "Grid" Graphics_. R package version 2.3, <https://CRAN.R-project.org/package=gridExtra>.
Hmisc	Harrell Jr F (2023). _Hmisc: Harrell Miscellaneous_. R package version 5.1-0, <https://CRAN.R-project.org/package=Hmisc>.
lme4	Douglas Bates, Martin Maechler, Ben Bolker, Steve Walker (2015). Fitting Linear Mixed-Effects Models Using lme4. *Journal of* S*tatistical* S*oftware*, 67(1), 1-48. https://doi.org/10.18637/jss.v067.i01.
lmerTest	Kuznetsova A, Brockhoff PB, Christensen RHB (2017). “lmerTest Package: Tests in Linear Mixed Effects Models”. _*Journal of* S*tatistical* S*oftware*_, ∗82∗(13), 1-26. https://doi.org/10.18637/jss.v082.i13 <https://doi.org/10.18637/jss.v082.i13>.
Lubridate	Garrett Grolemund, Hadley Wickham (2011). Dates and Times Made Easy with lubridate. *Journal of* S*tatistical* S*oftware*, 40(3), 1-25. URL https://www.jstatsoft.org/v40/i03/.
Mediation	Dustin Tingley, Teppei Yamamoto, Kentaro Hirose, Luke Keele, Kosuke Imai (2014). mediation: R Package for Causal Mediation Analysis. *Journal of* S*tatistical* S*oftware*, 59(5), 1-38. URL https://www.jstatsoft.org/v59/i05/.
Parallel	R Core Team (2023). _R: A Language and Environment for Statistical Computing_. R Foundation for Statistical Computing, Vienna, Austria. <https://www.R-project.org/>.
Purr	Wickham H, Henry L (2023). _purrr: Functional Programming Tools_. R package version 1.0.1, <https://CRAN.R-project.org/package=purrr>.
Rmisc	Hope RM (2022). _Rmisc: Ryan Miscellaneous_. R package version 1.5.1, <https://CRAN.R-project.org/package=Rmisc>.
RSQLite	Müller K, Wickham H, James DA, Falcon S (2023). _RSQLite: SQLite Interface for R_. R package version 2.3.3, <https://CRAN.R-project.org/package=RSQLite>.
Tidyr	Wickham H, Vaughan D, Girlich M (2023). _tidyr: Tidy Messy Data_. R package version 1.3.0, <https://CRAN.R-project.org/package=tidyr>.
Tidyverse	Wickham H, Averick M, Bryan J, Chang W, McGowan LD, François R, Grolemund G, Hayes A, Henry L, Hester J, Kuhn M, Pedersen TL, Miller E, Bache SM, Müller K, Ooms J, Robinson D, Seidel DP, Spinu V, Takahashi K, Vaughan D, Wilke C, Woo K, Yutani H (2019). “Welcome to the tidyverse”. _*Journal of Open* S*ource* S*oftware*_, ∗4∗(43), 1686. https://doi.org/10.21105/joss.01686 <https://doi.org/10.21105/joss.01686>.

**Table A3:** Life cycle analysis values

Damage category	Unit	Haemosol BP 5000 mL Product number 955426	Prismocitrate 18/0 Non-PVC (APAC)	Prism0cal EN/VN	SP 414: Prismaflex 5-Litre effluent bag	Auto-Effluent set (Prismax) 115370	Haemofilter set ST100 (Prismaflex)	Haemofilter set ST150
GWP100	kg CO_2_-eq	0.6008679	0.6109661	0.6008679	0.5985857	2.976586	6.224895	6.949951

GWP100 : Global warming potential over a 100-year period, a metric used to compare the warming effect of difference greenhouse gases over a 100-year period relative to carbon dioxide. Inputs used for the calculations are from global supply data of the raw materials and average global values of electricity usage for induction and moulding of the plastic, integrated with the above analyses.

**Table A4:** Cost and CO_2_ emissions, unit calculations

Item	Weight (total)	Purchase price (AUD)	CO_2_-equivalent per 24 h	Clinical waste weight (g)	Nonclinical waste weight (g)	Clinical waste cost (AUD)	Nonclinical waste cost (AUD)	Total waste cost (AUD)
AN69 ST100 set	1060.58	362.76	6.22	1060.58	0	3.71	0	3.71
AN69 ST150 set	1103.58	384.31	6.95	1103.58	0	3.86	0	3.86
Auto-effluent set	546.35	288.1	2.98	461.73	84.62	1.61	0.025	1.64
Effluent bags	139.87	7.88	0.60	113.87	26	0.40	0.008	0.40
Haemosol	110.69	26.36	0.60	0	110.69	0	0.03	0.03
Prism0cal B22	110.69	34.11	0.60	0	110.69	0	0.03	0.03
Prismocitrate	88.23	34.11	0.61	0	88.23	0	0.03	0.03

1. Weight per item.

2. Individual commercial prices.

3. LCA values.

4. Weight of waste.

5. Distinguishing clinical versus nonclinical waste in both weight and cost of disposal.

6. Total cost of waste per item.

**Table A5:** Results by year: circuit parameters, quantities of consumables, CO_2_-e/day, and costs

Variable	2017	2018	2019	2020	2021	2022	2023	2024
Total Patients, n	336	368	393	204	122	121	409	512
Proportion hospital A, %	69.6	67.9	59.5	93.1	96.7	63.6	72.9	71.4
Proportion PrisMax, %	0	0	0	0	0	99.17	100	100
Proportion citrate, %	53.9	62.2	70.2	74.0	76.2	70.2	71.9	70.1
Blood flow rate, mL/min	194.4 [149.6–199.8]	192.5 [132.1–199.6]	182.6 [129.9–199.3]	196.1 [162.8–199.9]	196.1 [171.3–199.9]	149.9 [129.9–195.1]	150 [130–195.2]	150.0 [130–189.4]
Effluent flow rate, mL/kg/hr	39.1 [31.1–44.0]	40.0 [32.7–45.0]	40.4 [35.4–45.0]	41.5 [38.0–44.6]	41.9 [36.9–45.0]	31.0 [28.7–35.2]	31.0 [28.1–35.2]	30.2 [27.5–33.6]
Net ultrafiltration rate, mL/kg/hr	1.5 [0.9–1.9]	1.6 [1.0–2.0]	1.5 [0.8–2.1]	1.7 [1.3–2.2]	1.7 [1.3–2.2]	1.5 [0.8–2.0]	1.5 [0.9–2.0]	1.5 [1.0–2.0]
Predilution rate, mL/hr	16.4 [5.6–19.6]	17.1 [12.4–20.0]	17.5 [14.8–19.8]	18.0 [15.6–20.1]	17.6 [15.3–20.1]	15.0 [13.7–17.1]	15.6 [13.3–17.6]	14.9 [13.0–17.1]
Postdilution rate, mL/hr	2.4 [2.0–2.8]	2.4 [2.0–2.8]	2.3 [2.0–2.8]	2.4 [2.1–2.7]	2.4 [2.0–2.7]	2.2 [1.9–2.6]	2.3 [1.9–2.7]	2.3 [1.9–2.7]
Dialysis rate, mL/hr	19.3 [18.4–22.8]	19.3 [18.0–22.4]	19.0 [16.9–20.4]	18.9 [18.2–20.2]	19.1 [18.0–20.3]	10.4 [9.5–15.8]	10.1 [9.5–14.7]	9.7 [9.4–14.5]
Circuit life, hours	26.7 [16.8–38.7]	27.0 [17.3–40.6]	26.8 [17.6–38.1]	27.4 [17.3–41.2]	28.4 [17.9–38.4]	32.6 [19.1–51.9]	34.3 [19.1–47.3]	35.2 [22.1–49.2]
Predilution bags per day, n	6.5 [2.9–7.8]	7.2 [5.9–8.0]	7.3 [6.3–8.0]	7.6 [6.4–8.0]	7.5 [6.4–8.0]	6.8 [6.2–7.6]	6.5 [6.0–7.6]	6.5 [6.0–7.4]
Postdilution bags per day, n	1.4 [1.2–1.8]	1.3 [1.1–1.7]	1.3 [1.2–1.7]	1.3 [1.1–1.6]	1.3 [1.1–1.6]	1.3 [1.1–1.6]	1.3 [1.1–1.5]	1.2 [1.1–1.5]
Dialysis bags per day, n	8.3 [7.0–9.6]	8.2 [7.0–9.5]	7.9 [7.1–9.1]	8.1 [6.9–9.2]	8.4 [7.1–9.4]	5.1 [4.4–7.4]	4.8 [3.9–7.2]	4.8 [3.9–7.1]
Effluent bags per day, n	15.3 [12.5–17.4]	16.1 [13.3–17.6]	16.4 [14.5–17.8]	16.7 [15.0–18.2]	16.6 [15.2–18.2]	11.3 [0.0–13.6]	11.1 [0.0–13.7]	11.1 [0.0–13.5]
Sets per day, n	0.9 [0.6–1.4]	0.9 [0.6–1.4]	0.9 [0.6–1.4]	0.9 [0.6–1.4]	0.8 [0.6–1.3]	0.7 [0.5–1.2]	0.7 [0.5–1.2]	0.7 [0.5–1.1]
Auto effluent per day, n	0.0 [0.0–0.0]	0.0 [0.0–0.0]	0.0 [0.0–0.0]	0.0 [0.0–0.0]	0.0 [0.0–0.0]	0.0 [0.0–0.5]	0.0 [0.0–0.4]	0 [0–0.4]
Predilution cost, $AUD	209.59 [75.77–266.99]	232.96 [156.65–270.65]	237.14 [200.57–267.91]	253.96 [208.30–272.40]	254.36 [203.66–273.44]	217.92 [188.31–257.47]	213.95 [188.80–252.24]	211.59 [190.80–241.21]
Postdilution cost, $AUD	42.4 [36.31–53.06]	40.84 [34.6–50.63]	40.73 [34.8–50.24]	42.16 [37.56–51.33]	41.76 [35.81–50.19]	38.86 [33.00–48.60]	38.84 [33.89–46.74]	38.48 [33.72–45.47]
Dialysis cost, $AUD	253.64 [210.15–294.67]	258.66 [220.07–299.14]	258.12 [219.28–293.19]	260.21 [221.42–303.03]	255.83 [226.16–307.84]	165.69 [134.48–238.85]	156.75 [124.38–235.54]	154.5 [123.69–217.64]
Effluent cost, $AUD	120.47 [98.25–137.14]	126.64 [105.09–138.36]	128.92 [114.52–139.92]	131.97 [117.78–143.61]	131.23 [120.02–143.82]	88.84 [0.00–107.32]	87.88 [0.00–107.90]	87.63 [0.00–106.35]
Sets cost, $AUD	344.51 [236.80–541.59]	336.16 [220.92–518.30]	336.8 [237.31–512.84]	336.17 [223.90–527.12]	325.12 [239.90–516.57]	283.18 [177.80–481.61]	268.51 [195.00–482.00]	261.88 [187.62–418.15]
Auto effluent cost, $AUD	0.00 [0.00–0.00]	0.00 [0.00–0.00]	0.00 [0.00–0.00]	0.00 [0.00–0.00]	0.00 [0.00–0.00]	0.00 [0.00–145.11]	0.00 [0.00–105.27]	0.00 [0.00–113.23]
Waste cost, $AUD	10.46 [8.95–12.51]	10.44 [8.92–12.44]	10.69 [9.44–12.86]	10.72 [9.22–12.83]	10.75 [9.53–12.52]	7.96 [6.02–9.92]	7.91 [6.42–10.07]	7.72 [6.34–9.54]
Total cost, $AUD	995.89 [820.40–1210.67]	992.48 [841.78–1200.82]	1024.25 [886.58–1230.87]	1037.93 [888.71–1232.12]	1027.44 [888.20–1217.06]	902.22 [750.88–1144.75]	832.32 [723.77–1102.70]	829.07 [689.64–1069.19]
Predilution CO_2_ emissions, CO_2_ equivalents per day	3.9 [1.7–4.8]	4.4 [3.5–4.9]	4.4 [3.8–4.9]	4.6 [3.9–4.9]	4.6 [3.9–4.9]	4.1 [3.8–4.7]	3.9 [3.6–4.6]	4.0 [3.6–4.5]
Postdilution CO_2_ emissions, CO_2_ equivalents per day	0.8 [0.7–1.1]	0.8 [0.7–1.0]	0.8 [0.7–1.0]	0.8 [0.7–1.0]	0.8 [0.7–1.0]	0.8 [0.7–0.9]	0.8 [0.7–0.9]	0.7 [0.7–0.9]
Dialysis CO_2_ emissions, CO_2_ equivalents per day	5.0 [4.2–5.8]	4.9 [4.2–5.7]	4.7 [4.2–5.5]	4.9 [4.2–5.6]	5.0 [4.2–5.6]	3.1 [2.7–4.4]	2.9 [2.4–4.3]	2.9 [2.4–4.3]
Effluent CO_2_ emissions, CO_2_ equivalents per day	9.1 [7.5–10.4]	9.6 [8.0–10.5]	9.8 [8.7–10.6]	10.0 [8.9–10.9]	10.0 [9.1–10.9]	6.7 [0.00–8.1]	6.7 [0.00–8.2]	6.7 [0.0–8.1]
Sets CO_2_ emissions, CO_2_ equivalents per day	6.1 [4.3–9.6]	6.1 [3.9–9.1]	6.0 [4.3–9.0]	6.1 [4.0–9.4]	5.9 [4.3–9.3]	5.1 [3.2–8.7]	4.9 [3.5–8.7]	4.7 [3.4–7.6]
Auto effluent CO_2_ emissions, CO_2_ equivalents per day	0 [0-0]	0.0 [0.0–0.0]	0.0 [0.0–0.0]	0.0 [0.0–0.0]	0.0 [0.0–0.0]	0.0 [0.0–1.0]	0.0 [0.0–0.5]	0.0 [0.0–0.5]
Total CO_2_ emissions, CO_2_ equivalents per day	25.3 [21.5–29.7]	25.3 [22.1–29.9]	26.0 [23.3–30.1]	26.4 [23.1–30.4]	26.5 [23.8–30.0]	20.4 [16.7–23.4]	19.6 [16.2–23.8]	19.3 [16.4–22.8]

**Fig. A5:**
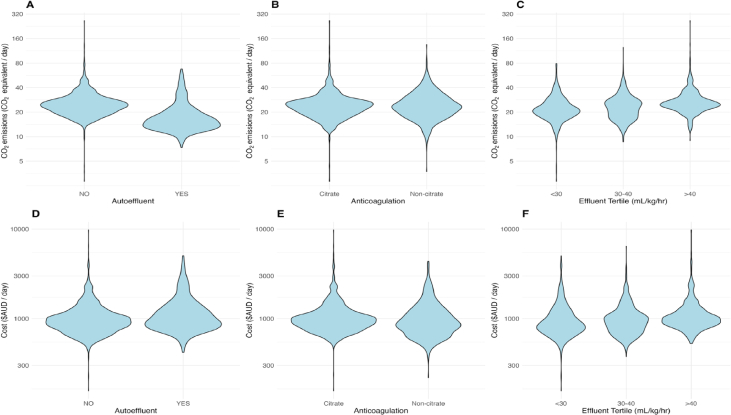
Violin plots of autoefflient, anticoagulation, effluent rate tertile versus CO_2_ emissions and cost 1. CO_2_ emissions for: A. Autoeffluent, B. Anticoagulation, C. Effluent tertile (ml/kg/hr) 2. Cost for D. Autoeffluent, E. Anticoagulation, F. Autoeffluent tertile (ml/kg/hr)

**Table A6:** Mediation models for citrate, filter life and CO_2_-e, and cost

CO_2_ equivalent				
Term	Estimate	95% CI	p value	Interpretation
ACME (indirect)	−0.099	[−0.120, −0.076]	<0.0001	Citrate reduces CO_2_ by prolonging filter life
ADE (direct)	+0.063	[+0.047, +0.079]	<0.0001	Citrate increases CO_2_ through other means
Total effect	−0.034	[−0.060, −0.008]	0.01	Net CO_2_ reduction, largely mediated by filter life
Proportion mediated	2.799	[1.81, 8.11]	0.01	280% mediated. Mediation dominates; direct and indirect effects in opposite directions

Cost				
*Term*	*Estimate*	*95% CI*	*p value*	*Interpretation*

ACME (indirect)	−0.127	[−0.155, −0.100]	<0.0001	Citrate reduces cost indirectly by extending filter life
ADE (direct)	+0.156	[+0.142, +0.170]	<0.0001	Citrate directly increases cost
Total effect	+0.029	[−0.004, +0.059]	0.08	Net effect not significant; opposing effects largely cancel
Proportion mediated	−3.904	[−31.2, +20.5]	0.08	High variability due to opposing direct and indirect effects, not significant.

ACME: average causal mediation effect; ADE: average direct effect; CI: confidence interval.

**Table A7:** Composition and weights of fluid bags, autoeffluent kit and outer packaging; indication of quantity of nonhazardous versus hazardous waste

Component/material	Weight in grams						
	Bag	Connections/ports/tubing	Outer packaging	Caps/clamps	Miscellaneous	Total hazardous waste	Total nonhazardous waste
*Prism0cal B22*							
Polypropylene	55.62		47.64 (inner layer)				
Polyamide			6.6 (outer layer)				
Polypropylene		6.6 (outer layer of injection port + 2 connections to patient)					
Polyethylene		0.2 (interface of inner and outer layers)					
Polycarbonate		0.63 (inner layer of injection port)					
Total							110.69
*Haemosol*							
Polypropylene	55.62		47.64 (inner layer)				
Polyamide			6.6 (outer layer)				
Polypropylene		6.6 (outer layer of injection port + 2 connections to patient)					
Polyethylene		0.2 (interface of inner and outer layers)					
Polycarbonate		0.63 (inner layer of injection port)					
Total							110.69
*Prisomocitrate 18/0*							
Polyamide			6.6 (outer layer of packaging)				
Polypropylene	42.51 (irrigation bag + connectors)		39.12 (inner layer of packaging)				
Total							88.23
*Autoeffluent*							
PVC (polyvinyl chloride)		137.59 (tubing, line junctions)					
Polyethylene			7.65 (printed packaging)				
MBS		1.24 (connector)					
PET (polyethylentherephthlate copolyester)			76.97 (packaging)		150.73 (casette)		
PAA				0.9 (caps)			
Polypropylene				30.6 (clamps)			
PCT	135.56						
Paper					1.33 (adhesive strip)		
Polyethylene					3.78 (support for line)		
Total						461.73	84.62
*Effluent*							
Low-density polyethylene (fossil fuel based)			5.4 (clear packaging)				
High density polyethylene (fossil fuel based)				0.39 (caps)			
Paper			6.54 (printer packaging and printed sticker)				
Polypropylene		8.85 (connectors)	5.4 (clips)				
Polyamide			6.6 (packaging)				
PP-coated cardboard			1.67 (instructions for clip)				
PVC (polyvinyl chloride)	99.62 (effluent bag, connectors, and upper pipe—small proportion of rubber)						
Total						113.87	20.6

Values obtained during composition analysis at Melbourne University Healthcare Carbon lab.

Individual components separated and analysed using Fourier transform infrared spectroscopy, including attenuated total reflectance on Bruker ALPHA II compact FT-IR Spectrometer, to generate absorbance spectrograms (Figure A3 earlier).

**Table A8:** Predicted cost and CO_2_-e with increasing effluent dose

Effluent dose (mL/kg/hr)	Predicted daily cost ($)	Cost 95% CI	Predicted CO_2_-e (kg/day)	CO_2_-e 95% CI
10	705.4	[345.5] to [1406.2]	18.5	[10.2] to [32.7]
15	758.4	[372.2] to [1531.4]	20.5	[11.5] to [35.8]
20	815.7	[412.2] to [1637.9]	22.4	[12.6] to [38.5]
25	866.8	[438.3] to [1655.9]	23.7	[13.8] to [40.4]
30	905.7	[453.1] to [1814.7]	24.7	[13.8] to [42.4]
35	924.6	[455.5] to [1827.2]	25.63	[15.2] to [45.3]
45	987.4	[495.5] to [1968.1]	28.1	[15.8] to [51.5]
50	1003.2	[501.6] to [2043.1]	28.2	[16.0] to [51.2]

CI: confidence interval.
